# Modular Platform for Recombinant Production of Highly Toxic Bacterial Proteins

**DOI:** 10.1111/1751-7915.70453

**Published:** 2026-10-16

**Authors:** Rina Fraenkel, Inbar Cahana, Tomer Sivan, Tal Fisher, Hen Nadav, Shani Bruchim, Jenna Weisz, Noam Deouell, Shani Cheskis, Maor Shalom, Lionel Imbert, Yaara Oppenheimer‐Shaanan, Asaf Levy, Netanel Tzarum

**Affiliations:** ^1^ Department of Biological Chemistry, Alexander Silberman Institute of Life Sciences, Faculty of Mathematics & Science The Hebrew University of Jerusalem Jerusalem Israel; ^2^ The Department of Plant Pathology and Microbiology, Institute of Environmental Sciences, Robert H. Smith Faculty of Agriculture, Food, and Environment The Hebrew University of Jerusalem Rehovot Israel; ^3^ Centre National de la Recherche Scientifique (CNRS), the Commissariat à l'Énergie Atomique et aux Énergies Alternatives (CEA), Institut de Biologie Structurale (IBS) University Grenoble Alpes Grenoble France; ^4^ Department of Life Sciences Achva Academic College Arugot Israel; ^5^ The Center for Nanoscience and Nanotechnology The Hebrew University of Jerusalem Jerusalem Israel

## Abstract

Bacterial protein toxins constitute a vast, largely untapped reservoir of potent bioactive molecules for antimicrobial discovery and biotechnology. However, their intrinsic toxicity often prevents recombinant expression in microbial hosts, limiting biochemical and functional characterization. Here, we present a modular platform for the recombinant production of highly toxic bacterial proteins based on covalent fusion of polymorphic toxins (PTs) to their cognate immunity proteins (PIMs), enabling transient toxin neutralization during expression. This strategy enabled recombinant production and purification of three previously refractory toxin families, PT7, PT8 and PT9. We further streamlined the workflow by introducing intracellular protease‐mediated cleavage of the toxin‐immunity fusion, reducing purification from four steps to two and increasing the recovered yield of PT9 from approximately 1–2 mg/L to ~4 mg/L. Extending the approach to PT37 further showed that computational redesign of the immunity protein and fusion architecture can enable production when the native fusion is insufficient. Purified PT7 and PT8 retained nuclease activity following denaturation and refolding, while refolded PT9 was structurally validated by X‐ray crystallography, demonstrating recovery of functionally and structurally competent proteins. Together, these findings establish a modular strategy for recombinant production of highly toxic bacterial proteins and facilitate their biochemical and functional characterization.

## Graphical abstract

A modular recombinant production platform based on transient toxin neutralization enables scalable expression and purification of intrinsically toxic proteins. The approach combines natural toxin–immunity interactions with computational protein engineering to expand production of previously intractable proteins.

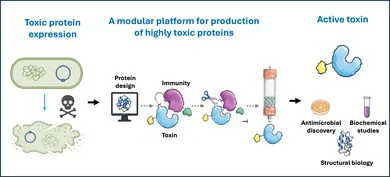

## 1 Introduction

Antimicrobial‐resistant (AMR) bacterial pathogens caused an estimated 4.95 million deaths worldwide in 2019, including 1.27 million deaths directly attributed to bacterial AMR (Antimicrobial Resistance 2022). In parallel, invasive fungal diseases represent an additional and growing global health burden, causing approximately 6.5 million infections and 3.8 million deaths annually, with 2.5 million deaths directly attributable to fungal infections (Denning 2024). The limited number of antifungal drug classes, together with the rapid emergence of drug‐resistant fungal strains, further exacerbates this crisis. Without effective intervention, AMR‐associated mortality is projected to reach 10 million deaths annually by 2050, prompting the World Health Organization (WHO) to designate AMR as one of the top global public health threats (WHO 2022).

The rapid global spread of multidrug‐resistant bacterial and fungal pathogens underscores the urgent need for antimicrobial agents with novel mechanisms of action (MoAs) that are less likely to develop resistance. However, discovering and developing new antimicrobial compounds faces major scientific, economic and regulatory challenges, resulting in a diminishing clinical pipeline (Gigante et al. 2024). Antifungal drug development is particularly challenging because fungal pathogens, as eukaryotes, are closely related to humans, limiting the availability of selective therapeutic targets and increasing the risk of patient toxicity (Roemer and Krysan 2014; Roy et al. 2023; Li et al. 2025; Souza et al. 2025). These limitations highlight the need to explore alternative classes of antimicrobial agents with distinct biological activities and mechanisms.

Among the most promising alternatives to conventional antibiotics are naturally occurring antimicrobial proteins, a diverse class of molecules that mediate microbial competition across all domains of life. These proteins employ a wide range of mechanisms to eliminate competing microorganisms, including disruption of membranes, inhibition of cell‐wall biosynthesis, interference with protein synthesis and degradation of nucleic acids (Zhang and Gallo 2016; Lei et al. 2019; Mookherjee et al. 2020). Their therapeutic potential is exemplified by the clinical success of peptide‐ and protein‐based antimicrobials such as polymyxins, gramicidin, nisin and echinocandins (Guiotto et al. 2003; Zheng et al. 2025). Among these molecules, antimicrobial small proteins, typically 50–150 amino acids in length, represent a particularly underexplored class. Many antimicrobial small proteins function as compact enzymatic toxins that disable essential cellular processes with remarkable potency. A prominent example is the bacterial polymorphic toxin (PT) system, which consists of modular proteins carrying highly diverse toxic C‐terminal effector domains that target essential cellular processes, including nucleic acid metabolism, protein synthesis and cell‐wall biogenesis (Zhang et al. 2012; Jamet and Nassif 2015; Ruhe et al. 2020). To prevent self‐intoxication, toxin‐producing bacteria co‐express highly specific cognate immunity proteins that bind and neutralize their corresponding toxin domains. Their exceptional potency, mechanistic diversity and modular organization make PTs attractive candidates for next‐generation antimicrobial therapeutics and valuable tools for discovering previously unknown antibacterial mechanisms.

Despite their considerable biological and therapeutic potential, recombinant production of enzymatic antimicrobial toxins remains a major unsolved challenge. Recombinant protein production is routinely performed in *Escherichia coli* ( *E. coli* ) because of its rapid growth, low cost and scalability (Rosano and Ceccarelli 2014). However, unlike most recombinant proteins, antimicrobial toxins directly attack highly conserved bacterial processes required for protein synthesis and cell viability (Baneyx 1999). Consequently, even low levels of basal expression frequently impose strong selective pressure for plasmid loss or inactivating mutations while simultaneously impairing protein accumulation through growth inhibition, proteolytic degradation, aggregation and inclusion‐body formation (Sorensen and Mortensen 2005; Rosano and Ceccarelli 2014; Hagan et al. 2023). Existing recombinant production strategies‐including tightly regulated promoters, co‐expression of immunity proteins, inclusion‐body purification and cell‐free expression are often protein‐specific, labour‐intensive, costly, or difficult to scale. As a result, recombinant production remains the principal bottleneck preventing systematic biochemical, structural and mechanistic investigation of these proteins.

An additional essential step in developing antimicrobial toxins into effective therapeutics is understanding their MoAs. Mechanistic characterization elucidates the structural and functional determinants that govern potency, specificity and stability and enables rational engineering to improve efficacy and minimize the emergence of resistance (Raheem and Straus 2019; Li et al. 2022). Nevertheless, many toxin proteins remain mechanistically uncharacterized, representing a vast, largely untapped reservoir of novel antimicrobial activities. Systematic discovery of novel toxin proteins and their unique MoAs could uncover fundamentally new strategies to combat bacterial and fungal pathogens and overcome existing resistance mechanisms (Nachmias et al. 2024). Realizing the therapeutic and biotechnological potential of these highly toxic proteins critically depends on scalable, tightly controlled bioproduction platforms that enable efficient recombinant expression and manufacturing.

Recent advances in genomics and high‐throughput bioinformatics provide unprecedented opportunities to systematically identify previously unrecognized antimicrobial toxin proteins and investigate their biological activities. Leveraging these approaches, we developed a computational framework to identify novel antimicrobial toxin proteins and applied it to analyse more than 100,000 bacterial genomes (Nachmias et al. 2024). This effort led to the discovery of nine previously uncharacterized toxin domains within polymorphic toxin systems (PT1‐PT9), each representing a distinct protein family defined by sequence similarity (Zhang et al. 2012; Ruhe et al. 2020). For a subset of PTs, we identified their associated immunity genes (Polymorphic Immunity genes, PIMs) that protect the toxin‐producing microorganism from self‐toxicity (Aoki et al. 2010). Functional validation confirmed strong antibacterial activity against *E. coli* and, for several PTs, demonstrated strong antifungal activity against multiple fungal pathogens, including top‐priority human pathogens listed by the WHO.

The identification of these antimicrobial toxins created an opportunity to explore a new class of antimicrobial proteins. However, realizing their scientific and translational potential requires scalable production of purified, biologically active proteins. Because conventional recombinant expression repeatedly failed for these highly toxic proteins (Nachmias et al. 2024), developing a robust, scalable and broadly applicable production platform became the critical next step towards their biochemical characterization and future biotechnological exploitation.

Here, we address this challenge by establishing a modular production platform based on transient intramolecular neutralization of toxin activity during recombinant expression. By integrating toxin‐immunity fusion proteins, streamlined purification and computational protein engineering, our approach overcomes key bottlenecks in the recombinant production of highly toxic proteins. We demonstrate the platform using multiple PTs that were previously refractory to recombinant expression and show that the recovered proteins retain their native nuclease activity. By purifying the wt PTs we could show that a previously identified PT (PT8) acts as a novel nuclease and could solveenabled solving the crystal structure of PPT9‐PIM9 complex. Collectively, this work establishes a microbial biotechnology platform for producing intrinsically toxic proteins and provides a foundation for their systematic biochemical, structural and translational investigation.

## 2 Materials and Methods

### 2.1 Plasmids Construction

The plasmids used in this project are as follows: the pET28a(+) vector (Novagen) was used for IPTG‐inducible expression. This vector contains a T7 promoter for high‐level expression and a pBR322 origin of replication (Ori). The pBAD24 vector (Thermo Fisher Scientific catalogue number V43001) was used for tightly regulated arabinose‐inducible expression. The vector contains the araBAD promoter, which enables low basal expression and tightly controlled induction conditions and a pBR322 Ori. pBAD24‐p15Aori is a modified pBAD24 in which the pBR322 Ori was replaced with the p15A origin (Figure S3a). The pETDuet‐1 vector (Novagen) was used for co‐expression experiments involving toxin‐immunity protein pairs. This dual‐expression vector contains two multiple cloning sites, each driven by independent T7 promoters and ribosome‐binding sites, enabling simultaneous expression of two recombinant proteins. The pIVEX2.4d vector was used for bacterial cell‐free protein expression experiments. This vector is optimized for coupled transcription‐translation reactions in *E. coli* ‐based cell‐free lysates and contains a T7 promoter for in vitro protein synthesis.

Plasmids were constructed using the Gibson Assembly method (Gibson et al. 2009). Vector backbones were linearized by PCR using primers flanking the multiple cloning site (Sigma‐Aldrich, Israel). PT and PIM genes were amplified from plasmids generated in our previous study (Nachmias et al. 2024), incorporating either a C‐terminal 6 × His tag (PTs) or an N‐terminal Strep tag (PIMs). Genes encoding the FusionPT constructs were synthesized commercially (Twist Bioscience). The pET28‐MBP‐GFP plasmid was constructed by inserting the MBP‐TEV site‐GFP sequence with a C‐terminal 6XHis Tag into the multiple cloning site of the pET28 plasmid.

DNA fragments were designed with 15–20  base pairs overlapping regions and assembled using Gibson Assembly. Reactions were performed in a total volume of 4 μL containing 50 ng of linearized vector and an equimolar amount of insert DNA. Assembly mixtures were incubated at 50°C for 1 h and subsequently transformed into chemically competent E. coli TOP10 cells by heat shock. Transformants were selected on LB agar supplemented with 2% glucose and the appropriate antibiotic, kanamycin for pET28‐derived plasmids and ampicillin for pBAD‐, pETDuet‐1‐ and pIVEX2.4d‐derived plasmids. Individual colonies were screened and verified by Sanger sequencing.

### 2.2 Construction of the pBAD‐MBP‐TEVP‐p15A Vector and Generation of tBL21‐TEVP Competent Cells

A synthetic DNA fragment encoding maltose‐binding protein (MBP) followed by a TEV cleavage site and fused to tobacco etch virus protease (TEVP) was inserted into the multiple cloning site of pBAD‐p15A (pBAD‐MBP‐TEVP‐p15A vector, hereafter pBAD‐TEVP) (Figure S3a). To generate transient TEVP‐expressing BL21(DE3) competent cells (tBL21‐TEVP), chemically competent BL21(DE3) cells were transformed with pBAD‐TEVP and selected on LB agar supplemented with ampicillin. Individual transformants were used to prepare chemically competent tBL21‐TEVP cells.

### 2.3 Small‐Scale Expression of the FusionPT Proteins

Chemically competent BL21(DE3) cells were transformed with the target plasmid and selected on LB agar supplemented with the appropriate antibiotic. For small‐scale expression, overnight culture was grown in LB medium supplemented with 2% glucose and the appropriate antibiotic. The next day, the culture was diluted 1:100 into 5 mL of fresh medium supplemented with the appropriate antibiotic and 0.1% glucose, incubated at 37°C until OD_600_ ≈ 0.5–0.6, and the inducer (1 mM IPTG or 0.2% arabinose) was added. After 2 h, 4 h, or overnight incubation at 23°C, 30°C, or 37°C, 0.5 mL samples were harvested by centrifugation, then lysed with BugBuster Master Mix (Millipore, Cat. No. 71456) supplemented with 1 mM PMSF (Sigma‐Aldrich, Israel) and Protease Inhibitor Cocktail Set V (Millipore, Cat. No. 539137). Soluble and insoluble fractions were separated by centrifugation and analysed by SDS‐PAGE.

### 2.4 Protein Expression and Purification

For preparative protein expression (1–2 L), cultures were grown and induced as described above for small‐scale expression. Cells were harvested by centrifugation and resuspended in lysis buffer consisting of 20 mM Tris–HCl (pH 7.5), 100 mM NaCl, 5 mM MgCl_2_ and 0.2% Triton X‐100 supplemented with lysozyme and protease inhibitors. Cells were disrupted using a Cell Disruptor (Microfluidics LV1‐1), and insoluble material was removed by centrifugation for 30 min at 4°C.

For the standard fusion protein purification workflow, clarified soluble lysates were loaded onto a 3‐ml Strep‐Tactin Sepharose column (Cytiva) pre‐equilibrated with buffer containing 20 mM Tris–HCl (pH 7.5) and 100 mM NaCl (Tris buffer) using an ÄKTA chromatography system. Following washing, bound proteins were eluted with phosphate‐buffered saline (PBS) supplemented with 2.5 mM desthiobiotin (Cayman Chemical, 30418‐500). Fusion proteins were subsequently incubated overnight at 4°C with recombinant TEV protease at an enzyme‐to‐substrate molar ratio of approximately 1:20 to cleave the linker separating the PIM and PT domains. The next day, TEV cleavage was verified by SDS‐PAGE. Cleaved samples were denatured in 8 M urea for 3 h at room temperature and applied to a 5‐ml HisTrap FF column (Cytiva). The column was washed with denaturing buffer to remove contaminants. Refolding of immobilized PTs was achieved by gradually replacing the denaturing buffer with Tris buffer directly on the column. Proteins were eluted with the Tris buffer supplemented with 250 mM imidazole. Eluted proteins were concentrated and further purified by SEC using a Superdex 75 or Superdex 200 column (Cytiva) equilibrated with Tris buffer. Fractions corresponding to the expected elution volume were pooled, concentrated, flash‐frozen and stored at −80°C.

### 2.5 Optimized Purification Workflow Based on Intracellular Proteolysis

For the optimized purification workflow, intracellular TEV‐mediated cleavage was performed during protein expression, eliminating the need for Strep‐tag affinity purification and in vitro protease treatment. For protein expression, the fusion‐containing plasmid was transformed into chemically competent tBL21‐TEVP cells. Protein expression was performed as described above, except that IPTG and arabinose were added simultaneously to induce expression of both the fusion protein and TEV protease. Following cell lysis, soluble fractions were incubated in 8 M urea for 3 h and directly applied to a 5‐ml HisTrap FF column (Cytiva). Bound proteins were washed under denaturing conditions and subsequently refolded on‐column by gradual exchange into the Tris buffer. Proteins were eluted with Tris buffer supplemented with 250 mM imidazole, concentrated and further purified by SEC using a Superdex 75 or Superdex 200 column (Cytiva). Fractions corresponding to monomeric protein were pooled, concentrated, aliquoted and stored at −80°C.

### 2.6 Protein Detection by Western Blot

Proteins were separated by SDS‐PAGE and transferred onto nitrocellulose membranes. Membranes were blocked, washed and incubated with the primary antibody, mouse anti‐His (LifeTein LLC LT0426), followed by incubation with HRP‐conjugated goat anti‐mouse secondary antibody (Jackson, 115–035‐062). Detection of Strep‐tagged proteins was performed using Precision Protein StrepTactin‐HRP Conjugate (Bio‐Rad, #1610381) according to the manufacturer's instructions. Next, membranes were washed and developed using enhanced chemiluminescence (ECL; azure biosystems, AC2101). Chemiluminescent signals were detected and recorded using a ChemiDoc imaging system (Bio‐Rad).

### 2.7 Nuclease and RNase Assay

To measure nuclease activity, 0.5 μg plasmid DNA (purified pAdVAntage plasmid; Promega) was incubated with PT7, PT8, PT9, DNase A (to a final nuclease concentration of 5 mM), or water (as a control) in reaction buffer containing 10 mM MgCl_2_, 100 μg/mL bovine serum albumin and 10 mM Bis‐Tris propane (pH 7) at 37°C for different time periods. Nuclease reactions were analysed on a 1.0% agarose gel and stained with ethidium bromide. RNase activity was measured under similar conditions using 0.5 μg of total ribosomal RNA extracted from mammalian cells (Rabany et al. 2026).

### 2.8 Drop Assay

Overnight culture was grown in LB medium supplemented with 2% glucose and the appropriate antibiotic. The next day, the culture was diluted 1:100 into fresh medium supplemented with the appropriate antibiotic and 0.1% glucose, incubated at 37°C until OD_600_ ≈ 0.5–0.6. Cultures were then serially diluted tenfold (10^0^–10^−8^) in sterile 96‐well plates, and 2 μL of each dilution was spotted in parallel onto repression plates (2% glucose) and induction plates (0.2% arabinose for pBAD or 0.01 mM IPTG for pET28a). Plates were incubated overnight at 37°C, and growth was documented the following day. Drop assays were repeated in three independent biological experiments with comparable results. Representative images are shown.

### 2.9 Cell Free

Cell‐free protein production: The proteins were produced in vitro in continuous exchange mode (CECF), with a reaction‐to‐dialysis volume ratio of 1:10. All reactions were performed at 27°C with gentle rotation at 18 rpm in a hybridisation oven (Hybrynogene K38, Ascon Technologic, Techne) using a 3 mL dialysis system (Maxi GeBaFlex, 8 kDa MWCO, Gene Bio‐Application Ltd) placed in 50 mL tubes containing 30 mL of feeding solution. The reaction mixtures contained the following: target protein pIVEX plasmid (16 μg/mL); *E. coli* BL21(DE3) S30 cellular extract; and T7 polymerase.

Other standard cell‐free components in the reaction and dialysis solutions (e.g., amino acids, NTPs, tRNAs, HEPES, MgOAc_2_, creatine phosphate, creatine kinase, NH_4_OAc, spermidine, cAMP, folinic acid) were added as described in a previously published protocol (Imbert et al. 2021). The cell‐free reaction was stopped after 16 h. The reaction mixtures were then centrifuged for 20 min at 14,000 g prior to western blot analysis.

### 2.10 Computational Redesign of the PT37‐PIM37 Fusion

The predicted structure of the FusionPT37 complex was used as the input backbone for sequence redesign with ProteinMPNN (Dauparas et al. 2022). ProteinMPNN was run using the default parameters, with a sampling temperature of 0.1, the v_48_020 model, one sequence generated per target (num_seq_per_target = 1) and a batch size of 1. Two design strategies were evaluated. In the first, the PT37 sequence was fixed, while the PIM37 and linker sequences were allowed to vary. In the second, both PT37 and the linker were fixed, restricting sequence redesign to PIM37. The resulting designs had ProteinMPNN scores of 0.981 and 0.902, respectively. ProteinMPNN scores represent the average negative log‐probability of the designed sequence conditioned on the input backbone, with lower scores indicating greater sequence compatibility with the specified backbone.

Both computationally designed candidates were subsequently evaluated experimentally. In addition, to assess the contribution of the redesigned linker independently of PIM37 redesign, we attempted to generate a construct containing native PIM37 and the redesigned linker. The sequences of the native and redesigned PIM37 and linker regions, including the introduced amino acid substitutions, are provided in Figure S4A,B.

### 2.11 Crystallization and Structural Determination of the PT9‐PIM9 Complex

For complex reconstitution, PIM9 was cloned into pET28 and expressed as an untagged protein using conditions similar to those used for the FusionPT constructs. Cell lysate containing PIM9 (from a 3 L LB culture) was mixed with 1.5 mg of purified, refolded PT9 and incubated for 4 h at room temperature with gentle agitation to allow complex formation. The PT9‐PIM9 complex was purified using a HisTrap HP column followed by SEC, as described above for the FusionPT constructs (Figure S5).

Crystallization of the PT9‐PIM9 complex was performed by the vapour‐diffusion sitting‐drop method at 20°C using a purified complex concentrated to 7 mg ml^−1^ in 20 mM Tris–HCl, pH 7.5, 100 mM NaCl. Initial crystallization plates were set up with the mosquito crystallization robot (SPT Labtech) using JCSGI‐IV (Molecular Dimensions). Crystals of the PT9‐PIM9 complex were obtained in reservoir solution containing 0.1 M Bicine pH 9.0 and 2.0 M Magnesium chloride. Crystals were cryoprotected in reservoir solution supplemented with 10%–15% ethylene glycol, mounted in loops and flash‐cooled in liquid nitrogen. X‐ray diffraction data were collected at 100 K at beamline ID30A‐3 of the European Synchrotron Radiation Facility (ESRF), Grenoble, France. Data were processed using XDS and scaled with AIMLESS. The structure was solved by molecular replacement in Phaser using a computational model of PT9‐PIM9 complex as the search model, followed by refinement in PHENIX (Adams et al. 2010), and model building was performed with COOT (Emsley and Cowtan 2004). Final refinement statistics are summarized in Table S1.

## 3 Results

### 3.1 Standard Recombinant Expression Methods Are Insufficient for Highly Toxic PT Proteins

Following the identification and functional validation of the PT families (Nachmias et al. 2024), we sought to express PT1^Em^ (PT1 from *Escherichia marmotae*, hereafter PT1), PT7^Bc^ (PT7 from *Bacillus cereus* , hereafter PT7), PT8^Li^ (PT8 from *Leptospira interrogans* , hereafter PT8) and PT9^Cn^ (PT9 from *Cedecea neteri* , hereafter PT9) as recombinant proteins for biochemical and structural characterization. These PT families were selected because they belong to the subset of toxins for which cognate immunity proteins have been identified (Nachmias et al. 2024), enabling evaluation of a toxin‐immunity fusion strategy for recombinant production. Because these toxins target essential bacterial processes and exhibit potent antibacterial activity, their recombinant production posed substantial technical challenges due to host toxicity.

The first step in all the expression strategies described below involves molecular cloning of the toxin genes into plasmids compatible with the intended expression system. Since cloning and plasmid amplification occur in bacteria, even low‐level basal toxin expression can be lethal to host cells, often resulting in plasmids carrying mutations within the toxin gene. To minimize basal toxin expression during cloning and plasmid propagation, all bacterial growth media used for molecular work were supplemented with 2% glucose. Glucose was used to repress basal expression from the arabinose‐inducible pBAD system and to reduce basal expression from the T7‐based pET system in BL21(DE3) cells. Despite these precautions, basal promoter leakage remained sufficient to select for inactivating mutations or to prevent stable propagation of several PT expression constructs, illustrating the exceptional toxicity of these proteins.

To evaluate recombinant expression, the genes encoding PT1, PT7, PT8 and PT9 were cloned as His‐tagged constructs into the arabinose‐inducible pBAD vector (see Materials and Methods). This vector exhibits relatively low basal expression and was previously used for toxicity validation assays of the PTs (Nachmias et al. 2024). In parallel, the genes were cloned into IPTG‐inducible pET28 vectors, which support high‐level protein production but exhibit higher basal expression. Transformation of the pBAD constructs into *E. coli* BL21(DE3) cells yielded colony numbers comparable to those obtained with control plasmids.

During initial optimization of the pET‐based expression system, several commonly used *E. coli* expression strains, including BL21(DE3), Rosetta(DE3), C43(DE3), pLysS(DE3) and HMS174(DE3), were evaluated for production of the wild‐type PTs. None of these strains resulted in detectable accumulation of recombinant PT under the conditions tested. We selected BL21(DE3) for subsequent experiments because it yielded the highest transformant recovery among the strains tested. Transformation of the pET28 constructs into BL21(DE3) revealed a strong effect of the wild‐type PTs on transformation efficiency. No colonies were recovered for PT1 across three independent transformations, compared with 1516 ± 76 colonies for the empty‐vector control. PT7, PT8 and PT9 yielded only 2.3 ± 0.5, 2.0 ± 1.0 and 93 ± 3.8 colonies, respectively (Figure S1A). Following induction, Western blot analysis showed no detectable accumulation of wild‐type PT7, PT8, or PT9 under the conditions tested (Figure S1B). Although all constructs were sequence‐verified before expression, we did not analyse the plasmid population after induction; therefore, we cannot exclude selection of genetic escape variants during culture or induction as an additional contributor to the lack of detectable protein accumulation.

Because co‐expression of cognate immunity proteins has been successfully used to enable the recombinant production of toxic bacterial effectors (Piscotta et al. 2019; Hagan et al. 2023), we next asked whether this strategy could overcome the expression barrier for PTs. We therefore applied this strategy to co‐express PT1, PT7, PT8 and PT9 with their cognate immunity proteins (PIMs). To facilitate co‐expression, toxin and immunity gene pairs were cloned into the dual‐expression vector pETDuet (see Materials and Methods). Initial cloning and expression attempts were largely unsuccessful: PT7‐PIM7 and PT9‐PIM9 could not be stably cloned, suggesting persistent toxicity, while PT1‐PIM1 and PT8‐PIM8 were successfully cloned but showed no detectable expression (Figure 1A,B). Next, we evaluated a co‐transformation strategy in which PT and PIM proteins were expressed from separate plasmids. PT8 and PT9 were cloned as His‐tagged proteins into a pBAD24‐p15Aori vector (modified pBAD24 with p15A origin, see Materials and Methods) to enable tightly regulated expression, while PIM8 and PIM9 were cloned as Strep‐tagged proteins into the pET28 vector. The pET28 plasmids encoding PIM8 or PIM9 were first transformed into BL21(DE3) cells, and the resulting strains were used to prepare competent cells. These competent cells were then transformed with the corresponding pBAD24‐p15Aori‐PT plasmid. To maximize toxin neutralization, we tested both simultaneous and sequential induction of the two proteins. In the sequential condition, PIM expression was induced for 2 h before the corresponding PT, allowing the immunity protein to accumulate prior to toxin production. Nevertheless, neither simultaneous induction nor PIM pre‐induction resulted in detectable accumulation of the PTs, despite detectable expression of the corresponding PIM proteins (Figure 1B,C). These findings indicate that co‐expression of cognate immunity proteins alone was insufficient to overcome the production barrier, suggesting that even transient accumulation of unbound toxin is sufficient to impair recombinant expression.

**FIGURE 1:**
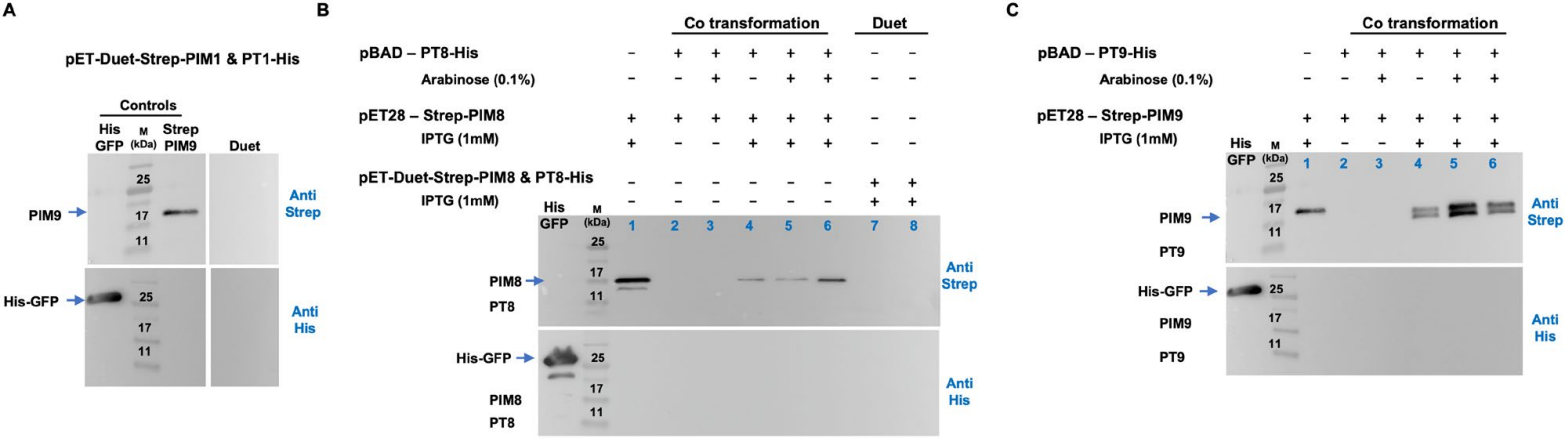
Expression of the PTs using standard recombinant expression approaches. (A) Co‐expression of His‐tagged PT1 and Strep‐tagged PIM1 from the dual‐expression vector pETDuet. Western blot analysis showed no detectable expression of PT1 or PIM1. The PT1 samples shown in (a) and the PT9 samples shown in (c) were run on the same gel and therefore share the same control lanes. (B) Co‐expression of His‐tagged PT8 and Strep‐tagged PIM8 using either co‐transformation of pBAD‐PT8 and pET‐PIM8 (Lanes 2–6) or the pETDuet vector (lanes 7–8). In co‐transformation experiments, PIM8 expression was induced either simultaneously with PT8 (Lane 5) or 2 h prior to PT8 induction (Lane 6). Western blot analysis showed no detectable PT8 expression under either condition. (C) Co‐expression of His‐tagged PT9 and Strep‐tagged PIM9 using co‐transformation of pBAD‐PT9 and pET‐PIM9. PIM9 expression was induced either simultaneously with PT9 (Lane 5) or 2 h before PT9 induction (Lane 6). Western blot analysis showed no detectable PT9 expression. PIM9 is occasionally detected as a double band under these expression conditions; the origin of the additional band is unknown.

Finally, we asked whether eliminating host‐cell viability constraints altogether could overcome the production barrier. Cell‐free (CF) expression bypasses host‐cell viability constraints, making it suitable for toxin expression (Ramm et al. 2024; Woelbern and Ramm 2024). PT1 could not be stably cloned into pIVEX plasmids for CF expression. In contrast, PT7, PT8 and PT9 were successfully cloned into pIVEX plasmids and expressed in the bacterial CF lysate. Western blot analysis showed no detectable accumulation of recombinant PTs (Figure S1C). These findings indicate that eliminating host‐cell viability constraints alone is insufficient for the recombinant production of these toxins, suggesting that the newly synthesized proteins directly interfere with the bacterial cell‐free transcription‐translation machinery before detectable protein accumulates.

Collectively, these experiments demonstrate that neither reducing basal expression, co‐expressing cognate immunity proteins, nor eliminating host‐cell viability is sufficient to enable recombinant production of these highly toxic PTs. These findings motivated the development of an alternative production strategy that maintains toxin neutralization throughout the recombinant expression process.

### 3.2 Fusion‐Based Strategy for the Production of Wild‐Type PTs

To overcome production barriers associated with highly toxic PTs, we developed a fusion‐based toxin neutralization strategy in which the PIM is covalently linked to the wild‐type (wt) PT within a single polypeptide chain. We reasoned that covalently linking the toxin and immunity protein would promote rapid intramolecular complex formation during translation, thereby minimizing transient accumulation of unbound cytotoxic toxin. The fusion construct is organized as follows: an N‐terminal Strep‐tagged PIM is connected via a flexible linker to a C‐terminal His‐tagged PT (Figure 2A). The linker comprises multiple Gly‐Gly‐Gly‐Ser (GGGS) repeats that provide conformational flexibility and facilitate formation of the predicted PIM‐PT interaction interface (described below). Following the linker is a Tobacco Etch Virus protease cleavage site (TEVcs), which enables precise separation of the two domains after purification. The overall workflow was designed to maintain toxin neutralization throughout recombinant expression and initial purification while enabling subsequent recovery of the isolated PT following proteolytic cleavage and affinity‐based purification. This strategy was intended to generate native‐like PTs suitable for downstream biochemical, structural and mechanistic analyses, as well as for future use as antimicrobials. TEV protease was selected because its highly specific cleavage generates the native PT with only a minimal N‐terminal scar (one amino acid), thereby minimizing potential effects on protein function. (Figure 2A) (Carrington and Dougherty 1988; Dougherty et al. 1989; Polayes et al. 1998). This minimal scar is particularly advantageous for downstream biochemical, structural and mechanistic studies, where additional N‐terminal residues can affect protein folding and activity.

**FIGURE 2:**
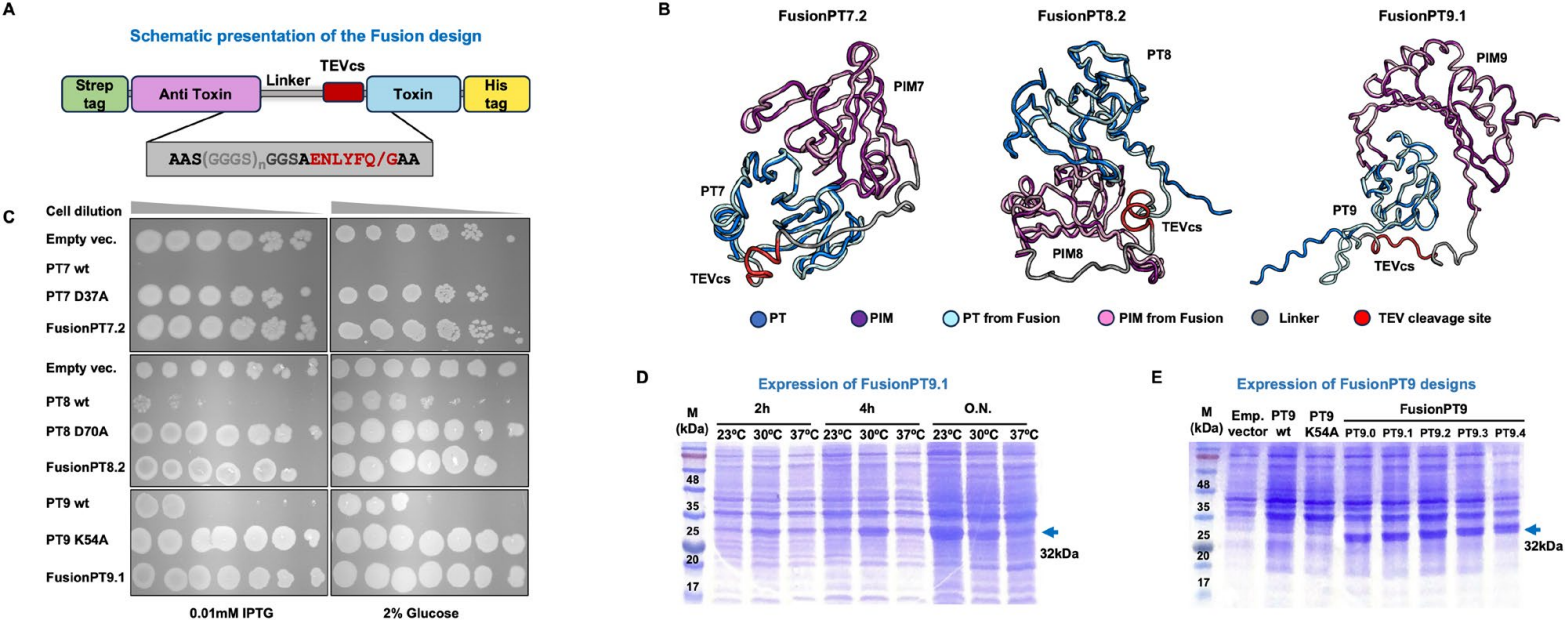
Structure‐guided design of FusionPT constructs. (A) Schematic illustration of the fusion‐based construct. The construct consists of an N‐terminal Strep‐tagged PIM linked to a C‐terminal His‐tagged PT through a flexible Gly‐Gly‐Gly‐Ser (GGGS) linker containing a Tobacco Etch Virus protease cleavage site (TEVcs). (B) The selected FusionPT constructs for PT7, PT8 and PT9 expression. Predicted structures of the native non‐covalent PT‐PIM complexes were superimposed on those of the FusionPT.0‐FusionPT.4 constructs containing GGGS linkers of varying lengths (Figure S2B). The optimal fusion design was chosen as the shortest linker that preserved the relative orientation of the PT and PIM domains while minimizing structural distortion. FusionPT7.2, FusionPT8.2 and FusionPT9.1 were selected for experimental validation. (C) Fusion‐based neutralization rescues PT toxicity. Drop assays of *E. coli* BL21(DE3) cells expressing FusionPT7.2, FusionPT8.2 and FusionPT9.1 under inducing conditions. Catalytically inactive PT mutants served as non‐toxic controls. Fusion protein expression restored bacterial growth to levels comparable to those of the inactive mutants, indicating efficient neutralization of PT toxicity by the covalently linked PIM. (D) Optimization of fusion protein expression conditions. SDS‐PAGE analysis of small‐scale FusionPT9.1 expression at different induction temperatures and expression times. Overnight expression at lower temperatures produced the highest protein yield and was used for subsequent purification experiments. (E) Effect of linker length on FusionPT9 expression. SDS‐PAGE analysis of FusionPT9 variants containing linkers of different lengths demonstrated comparable recombinant expression levels.

### 3.3 Structure‐Guided Design of PIM‐PTwt Fusion Proteins

Because no experimental structures of the PT‐PIM complex were available, linker design required computational prediction (e.g., Robetta, Boltz‐2 and AlphaFold2 (Kim et al. 2004, Jumper et al. 2021, Passaro et al. 2025)) of the relative orientation between the toxin and immunity protein. The linker must be sufficiently long to permit proper folding and intramolecular assembly of the PT‐PIM complex, while remaining short enough to avoid introducing an excessively flexible, structurally heterogeneous region.

We used Robetta (Kim et al. 2004) to model the non‐covalent PT‐PIM complexes using deep learning‐based structure prediction and generated a series of five fusion constructs containing one to five GGGS repeats designated FusionPT.0 through FusionPT.4, respectively (Figure S2A). Each construct was compared with the predicted native complex, and the shortest linker that preserved the native domain arrangement was selected for experimental validation. Based on this workflow, we designed fusion variants of PT7, PT8 and PT9 (Figures 2B and S2B). The same strategy was applied to PT1, for which five FusionPT1 constructs with different linker configurations were designed. However, PT1 proved exceptionally challenging already at the molecular cloning stage. Transformation yielded very few colonies, and only one of the five designs produced recoverable transformants. Sequencing of these colonies revealed frameshift mutations within the PT1 coding sequence, preventing recovery of a genetically intact FusionPT1 construct for subsequent expression studies.

Structural comparison of the predicted fusion constructs with their corresponding non‐covalent PT‐PIM complexes revealed that shorter linkers often imposed geometric constraints that distorted the relative orientation of the two domains (e.g., FusionPT9.0 and FusionPT7.0, Figure S2B), whereas longer linkers provided no substantial improvement in structural agreement. FusionPT7.2, FusionPT8.2 and FusionPT9.1 were selected for experimental validation.

To evaluate whether the fusion designs alleviate PT‐mediated toxicity during bacterial expression, we cloned the selected fusion constructs into the pET28 expression vector and transformed the resulting plasmids into *E. coli* BL21(DE3) cells. Toxicity was assessed by drop assay under inducing conditions. As controls, we included catalytically inactive PT mutants that have been shown to abolish toxicity and permit bacterial growth (Nachmias et al. 2024). Consistent with the proposed mechanism, expression of the fusion constructs restored bacterial viability to levels comparable to those of the non‐toxic PT mutants, demonstrating that covalent linkage of the PIM effectively neutralizes PT toxicity during expression (Figure 2C).

To further evaluate the fusion design strategy, we generated a series of FusionPT9 constructs with linkers of varying lengths (FusionPT9.0‐FusionPT9.4) and assessed toxicity using drop assays (Figure S2C). All fusion constructs restored bacterial growth. Notably, FusionPT9.1 consistently exhibited the strongest growth phenotype. These findings are consistent with the computational prediction that the shortest structurally compatible linker provides efficient intramolecular toxin neutralization.

### 3.4 Expression of FusionPT7.2, FusionPT8.2 and FusionPT9.1

In our previous study, several catalytically inactive PT mutants lost their toxic phenotype yet failed to accumulate as recombinant proteins (Nachmias et al. 2024). We therefore asked whether the fusion‐based neutralization strategy not only alleviates toxicity but also enables productive recombinant expression. In contrast to all conventional expression strategies tested above (Figure 1), small‐scale (5 ml of LB culture) induction readily produced detectable levels of FusionPT7.2, FusionPT8.2 and FusionPT9.1 proteins (Figure S2D), demonstrating that intramolecular toxin neutralization enables the recombinant production of these highly toxic PTs. We next optimized small‐scale expression conditions for subsequent preparative protein production. Overnight induction produced substantially higher protein yields than shorter induction periods and was therefore used for all subsequent purification experiments (Figures 2D and S2D). Comparison of the different FusionPT9 variants revealed no substantial differences in expression level, indicating that linker length primarily influences toxin neutralization (Figures 2C and S2C) rather than recombinant protein accumulation under the conditions tested (Figure 2E).

These findings demonstrate that intramolecular toxin neutralization not only restores bacterial viability but also overcomes the previously insurmountable barrier to recombinant production of these highly toxic proteins.

### 3.5 Purification of FusionPT7.2, FusionPT8.2 and FusionPT9.1

Having established expression of the fusion constructs, we next asked whether the fusion strategy could yield purified native toxins suitable for downstream biochemical and structural studies. To test this, the fusion proteins were purified, proteolytically cleaved to release the toxin domain, dissociated under denaturing conditions, and the isolated PTs were refolded and analysed by size‐exclusion chromatography (SEC). We first applied this workflow to FusionPT9.1. Strep‐trap affinity purification yielded high levels of soluble fusion protein, and SDS‐PAGE analysis indicated substantial enrichment of FusionPT9.1 after the affinity step (Figure 3A,B). Subsequent incubation with TEV protease resulted in complete cleavage of the fusion construct, generating the expected PIM and PT products (Figure 3B). To dissociate the cleaved PIM‐PT complex, the sample was incubated in 8 M urea and then loaded onto a Ni^2+^‐affinity column under denaturing conditions (Piscotta et al. 2019; Hagan et al. 2023). Because the PT domain retained its C‐terminal His‐tag, it could be selectively captured on the resin, while the dissociated PIM and other contaminants were removed during washing. Gradual urea removal directly on the column enabled refolding of the immobilized PT prior to elution (Figure 3C). Finally, the refolded protein was subjected to SEC, which revealed a predominantly monodisperse monomeric peak consistent with the successful recovery of soluble, properly folded PT9wt (Figure 3D). This procedure yielded highly purified PT9wt, as indicated by SDS‐PAGE analysis (Figure 3E).

**FIGURE 3:**
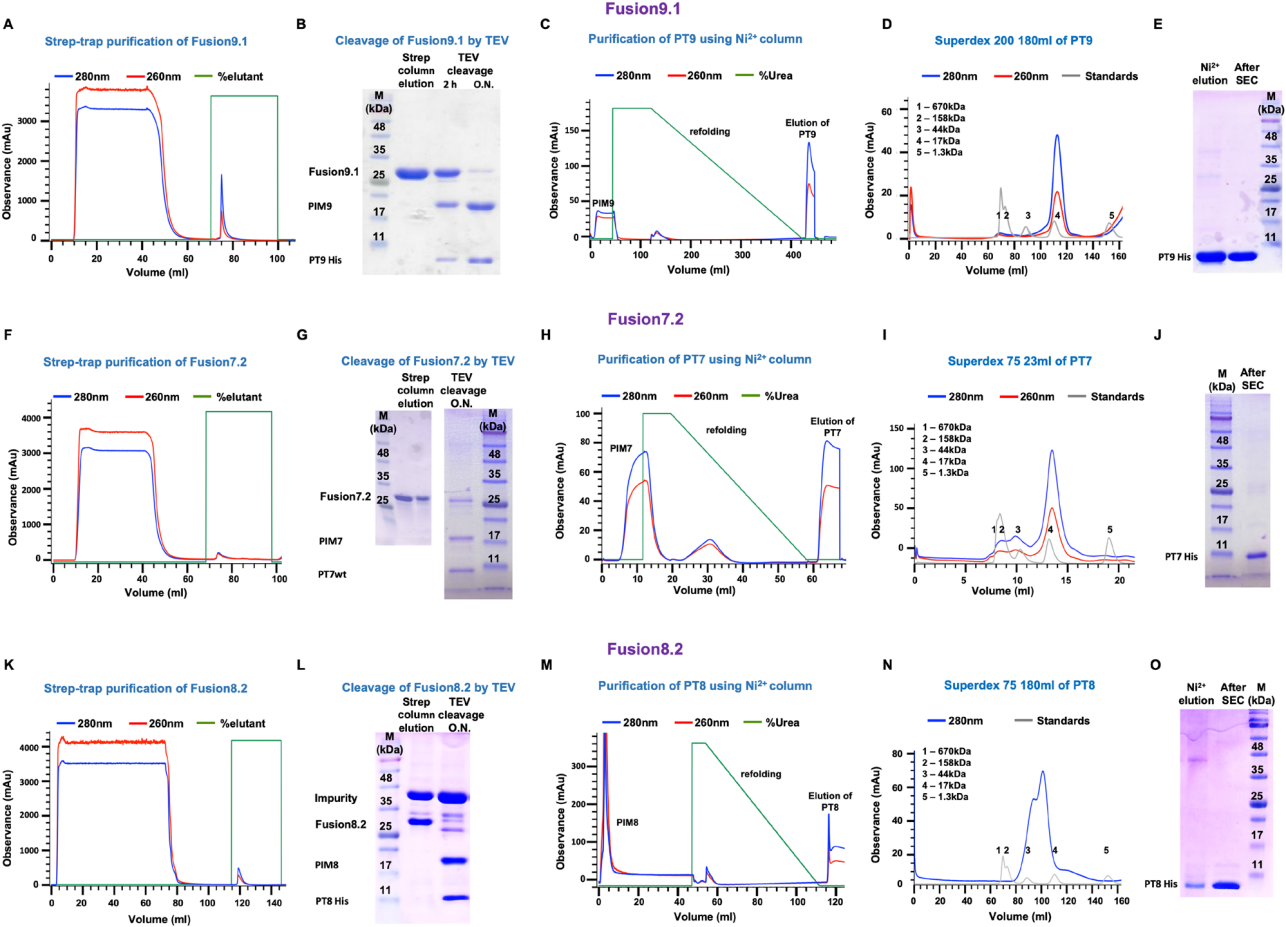
Purification of wild‐type PT7, PT8 and PT9 using the fusion‐based expression strategy. Purification of PT9 (A–E), PT7 (F–J) and PT8 (K–O). (A, F, K) Strep‐tag affinity purification of FusionPT9.1, FusionPT7.2 and FusionPT8.2. (B, G, L) In vitro TEV protease cleavage of the purified Fusion protein. Cleavage was assessed by SDS‐PAGE. FusionPT8.2 was co‐purifying with a ~ 50‐kDa contaminant; the contaminating protein remained uncleaved (L). (C, H, M) Purification of the wt PTs by Ni^2+^‐affinity chromatography following denaturation of the cleaved PIM‐PT complex in 8 M urea, on‐column refolding and elution under native conditions. (D, I, N) SEC profile of purified PTs. (E, J, O) SDS–PAGE analysis of the purified PTs following SEC indicating high purity.

Next, we applied the same workflow to FusionPT7.2. Strep‐tag affinity purification yielded substantial amounts of FusionPT7.2 (Figure 3F–J). TEV protease treatment resulted in near‐complete cleavage of the fusion construct (Figure 3G). Subsequent dissociation of the PIM‐PT complex under denaturing conditions, followed by Ni^2+^‐affinity purification and SEC, enabled recovery of highly purified PT7 (Figure 3H–J). Similarly, Strep‐tag affinity purification yielded substantial amounts of FusionPT8.2 protein (Figure 3K,L). Unlike PT7 and PT9, purification of FusionPT8.2 co‐purified an additional ~50‐kDa protein (Figure 3K,L). TEV protease treatment of the eluted proteins resulted in complete cleavage of FusionPT8.2, while the co‐purifying protein remained unaffected, consistent with its non‐specific origin (Figure 3L). Following dissociation of the PIM‐PT complex under denaturing conditions and Ni^2+^‐affinity purification, PT8 toxin was efficiently separated from the contaminating protein and recovered at high purity (Figure 3M–O).

Together, these results demonstrate that the fusion‐based purification strategy is robust across multiple PT families and enables recovery of highly purified wt toxins despite differences in purification behaviour and cleavage efficiency. Importantly, the strategy consistently yielded soluble proteins that could be successfully refolded after denaturation, providing material suitable for downstream biochemical and structural analyses.

### 3.6 Intracellular Proteolysis Simplifies and Improves the Fusion Production Platform

Having established that the fusion‐based strategy enables purification of multiple wt PTs, we next sought to simplify the workflow to improve scalability and reduce production costs. Although effective, the original purification protocol consisted of four steps performed over 3 days, including Strep‐tag affinity purification and in vitro TEV protease cleavage, both of which substantially increase production costs and processing time (Figure 4A). To address these limitations, we developed an optimized purification strategy based on intracellular TEV‐mediated cleavage of the fusion construct. We reasoned that the high affinity of the PT‐PIM interaction would maintain the complex after intracellular TEV cleavage, thereby preserving toxin neutralization while eliminating the need to purify the intact fusion protein and to perform subsequent in vitro proteolysis. This strategy reduced the workflow to two purification steps and eliminated the need for both Strep‐tag affinity purification and preparative TEV protease treatment (Figure 4A).

**FIGURE 4:**
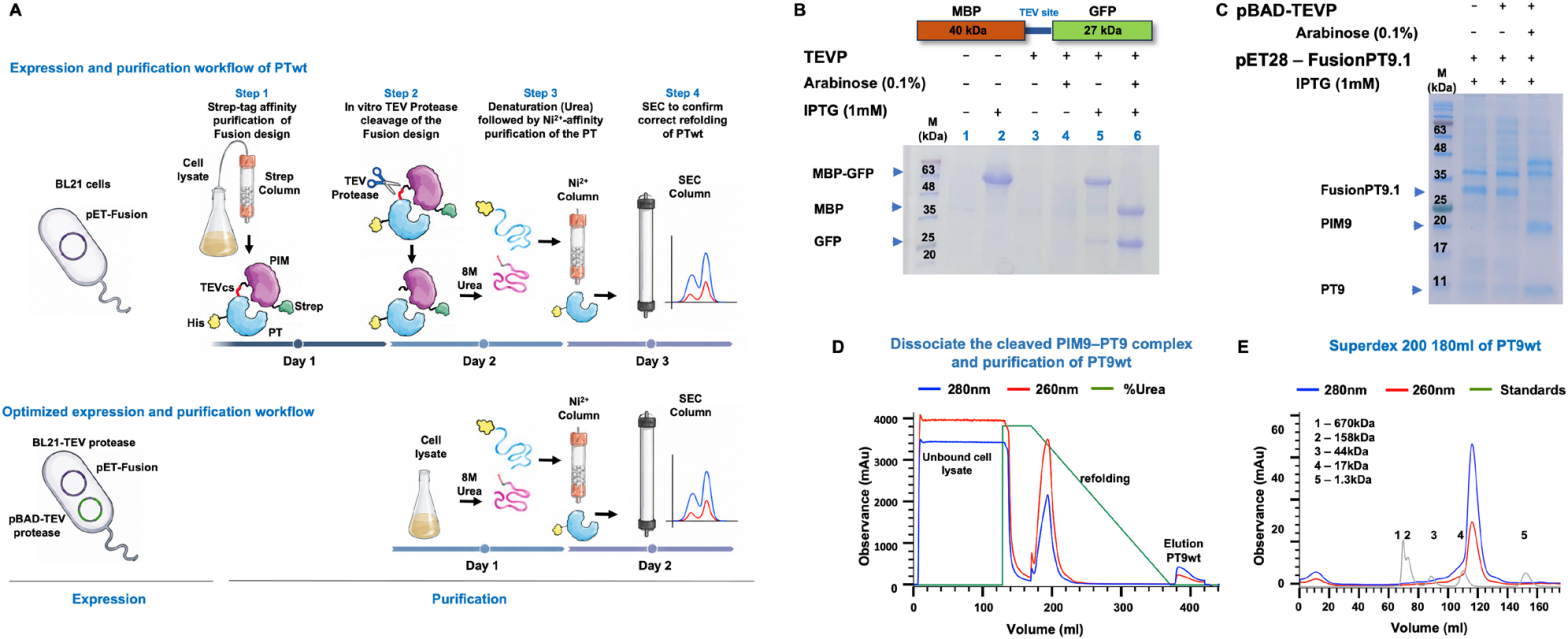
Optimization of the fusion‐based purification workflow through intracellular TEV‐mediated cleavage. (A) Schematic comparison of the original four‐step purification workflow and the optimized two‐step workflow. In the optimized protocol, intracellular TEV protease cleavage replaces both Strep‐tag affinity purification and in vitro TEV cleavage, enabling direct purification of the released PT by Ni^2+^ affinity chromatography, followed by SEC. (B) Validation of intracellular TEV protease activity using the MBP‐TEVcs‐GFP reporter. BL21(DE3) cells or tBL21‐TEVP cells were induced with IPTG alone or with IPTG and arabinose to co‐induce expression of MBP‐TEVcs‐GFP and TEV protease. SDS‐PAGE analysis demonstrates complete intracellular cleavage of the reporter protein upon TEV induction. (C) Intracellular cleavage of FusionPT9.1. FusionPT9.1 was co‐expressed with TEV protease in tBL21‐TEVP cells. SDS‐PAGE analysis demonstrates efficient cleavage of the fusion construct into the expected products. (D) Purification of PT9 using the optimized workflow. Following intracellular TEV‐mediated cleavage, soluble lysates were denatured in 8 M urea and PT9 was purified by Ni^2+^‐affinity chromatography with on‐column refolding. (E) SEC profile of purified PT9 obtained using the optimized purification workflow, showing recovery of a predominantly monodisperse protein consistent with successful refolding. SDS‐PAGE analysis confirms the high purity of PT9.

To enable intracellular cleavage, we engineered an arabinose‐inducible TEV protease expression plasmid compatible with the pET expression system by replacing the pBR322 origin of pBAD24 with the p15A origin (Figure S3A). The resulting pBAD‐TEVP plasmid was introduced into BL21(DE3) cells to generate the tBL21‐TEVP strain used throughout this study (Figure S3A).

To evaluate in‐cell proteolytic activity, tBL21‐TEVP competent cells were transformed with the pET28‐maltose‐binding protein (MBP)‐TEVcs‐GFP plasmids. As a control, the plasmid was transformed into BL21(DE3) cells. Induction of MBP‐GFP led to robust accumulation of the fusion protein in both strains (Figure 4B, wells 2 and 5). In contrast, co‐induction of TEV protease in tBL21‐TEVP cells resulted in complete cleavage of the MBP‐GFP fusion protein (Figure 4B, well 6). We next examined whether TEVP could efficiently cleave the FusionPT9.1 construct in vivo. To this end, pET28‐FusionPT9.1 was transformed into tBL21‐TEVP cells, and expression of both FusionPT9.1 and TEVP was induced simultaneously. SDS‐PAGE analysis revealed efficient cleavage of the fusion construct, yielding the expected cleavage products and demonstrating that intracellular TEV proteolysis is compatible with the FusionPT9 architecture (Figure 4C).

Having demonstrated efficient intracellular cleavage of FusionPT9.1, we next asked whether this simplified workflow could recover purified native PT9. FusionPT9.1 was co‐expressed with TEVP in tBL21‐TEVP cells, and the cells were then harvested and lysed. The soluble fraction was isolated and incubated with 8 M urea to dissociate the cleaved PIM‐PT complex. The denatured sample was loaded directly onto a Ni^2+^‐affinity column and gradually washed with buffer to promote refolding of the immobilized protein before elution (Figure 4D). SDS‐PAGE analysis showed high purity of PT9 (Figure S3B). Finally, SEC analysis revealed a predominantly monodisperse peak, consistent with successful refolding and recovery of soluble PT9 (Figure 4E). Importantly, in addition to reducing the purification workflow from four steps to two, the optimized protocol increased PT9 recovery from approximately 1–2 mg/L to ~4 mg/L of bacterial culture, representing a two‐ to four‐fold increase in protein yield.

Together, these results demonstrate that intracellular TEV‐mediated cleavage substantially simplifies the production platform without compromising purification efficiency. In addition to eliminating two purification steps and reducing reliance on affinity reagents and exogenous protease, the optimized workflow increased recombinant protein recovery, making the platform more suitable for production of highly toxic bacterial proteins.

### 3.7 Re‐design of Immunity Proteins Expands the Applicability of the Toxin Production Platform

Although the fusion‐based strategy enabled efficient production of multiple wt PTs, several PIM‐PT fusion constructs still failed to yield detectable recombinant protein. We hypothesized that, for these proteins, the native PIM does not bind its cognate toxin with sufficient affinity to fully suppress toxin activity during expression, allowing residual toxicity to impair recombinant protein production.

As a proof of concept, we selected PT37, a previously uncharacterized PT from *E. coli* that consistently failed recombinant production. To overcome this limitation, we sought to engineer improved toxin‐binding proteins through computational protein design. The approach was based on the structural features of the naturally occurring immunity proteins and sought to improve their interaction with the toxin domain.

Consistent with other highly toxic PTs, recombinant expression of PT37 yielded no detectable protein (Figure S4A), despite only a relatively mild toxic phenotype in drop assays (Figure 5A). We first applied our fusion‐based strategy by generating a FusionPT37 construct in which PT37 was fused to its predicted cognate immunity protein identified using AlphaFold2 (Jumper et al. 2021) (Figure 5B, left). Although the fusion construct rescued the toxic phenotype in drop assays (Figure 5C), no recombinant protein was detected in small‐scale expression experiments (Figure 5D, left), indicating that fusion to the native immunity protein was insufficient to support detectable recombinant PT37 production. We therefore investigated whether computational redesign of the immunity protein and fusion architecture could improve productive PT37 expression. ProteinMPNN (Dauparas et al. 2022), a structure‐based protein sequence design method that generates amino acid sequences compatible with a specified protein backbone, was used to redesign PIM37 and the fusion linker based on the predicted PT37‐PIM37 complex structure (Figure 5B). Two design strategies were evaluated. In the first, the PT37 sequence was fixed while both PIM37 and the linker were redesigned, generating DesFusionPT37 (Figure S4B,C). In the second, both PT37 and the linker were fixed, restricting redesign to PIM37 alone. The resulting designs had ProteinMPNN scores of 0.981 and 0.902, respectively (Figure S4B and Materials and Methods). Figure S4C shows the amino acid substitutions introduced into PIM37 and the linker.

**FIGURE 5:**
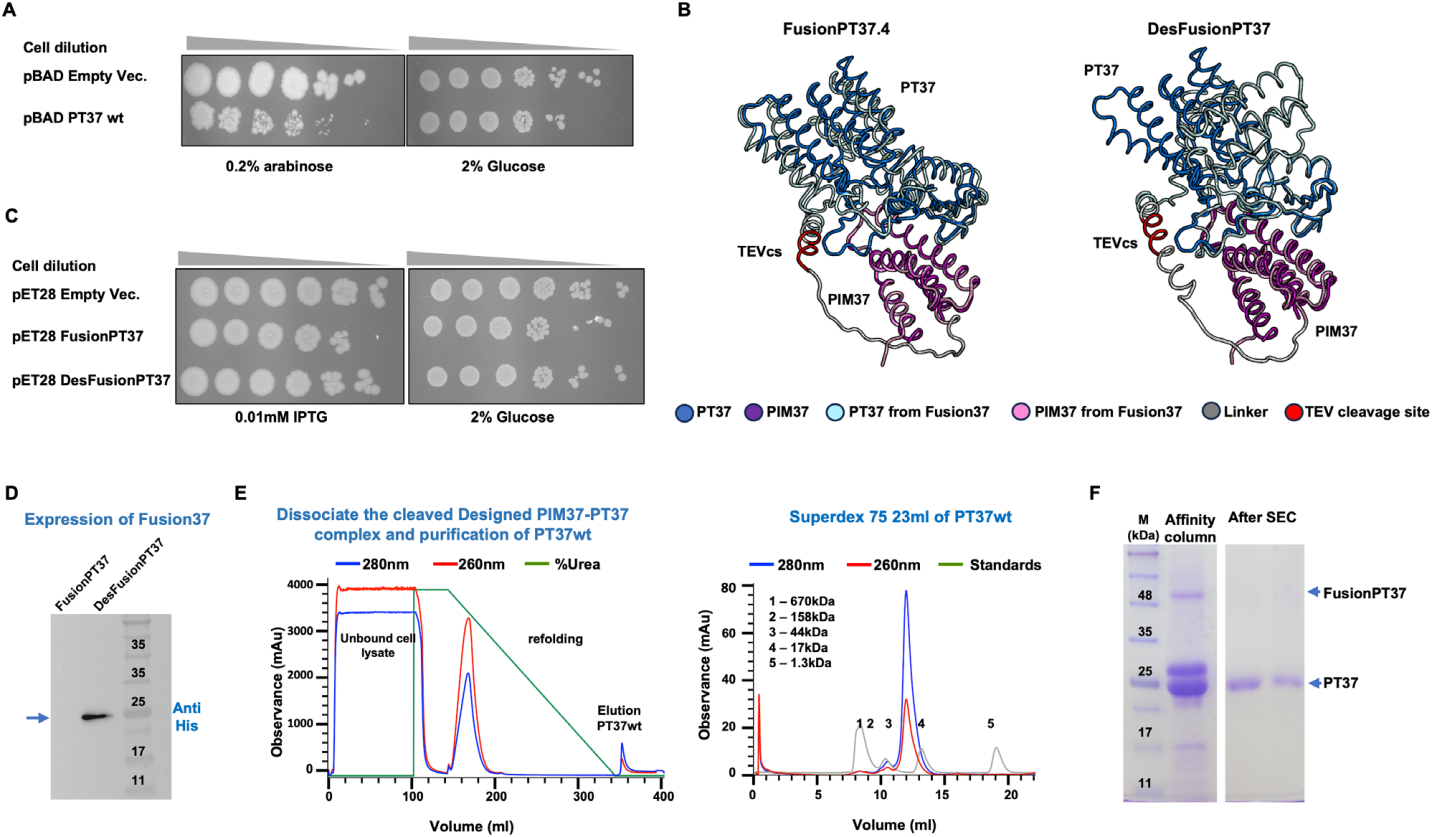
Computational redesign of immunity proteins expands the fusion‐based production platform. (A) Drop assay demonstrating the toxicity of PT37. (B) Structure‐guided design of the FusionPT37 and DesFusionPT37. Predicted structure of the native non‐covalent PT37‐PIM37 complex was superimposed with the corresponding FusionPT37 and DesFusionPT37 predicted structures. (C) Drop assay showing rescue of PT37 toxicity by FusionPT37 and DesFusionPT37. (D) Western blot analysis of small‐scale expression of FusionPT37 and DesFusionPT37 indicating recombinant expression of DesFusionPT37 but not FusionPT37. (E) Purification of PT37 using the optimized intracellular TEV‐mediated purification workflow. (F) SDS‐PAGE analysis of purified PT37.

To distinguish the contributions of the redesigned immunity protein and linker, we also attempted to generate a construct containing native PIM37 with the redesigned linker (Figure S4B–E). However, we could not recover a genetically intact construct. Sequencing of the resulting plasmid revealed deletion of the PT37 coding sequence. In contrast, constructs containing the redesigned PIM37 could be successfully cloned. Transformation of BL21(DE3) cells further revealed marked differences among the constructs. DesFusionPT37 produced substantially more transformants than the original FusionPT37 construct, whereas no transformants were recovered for the construct containing redesigned PIM37 with the native linker (Figure S4B). Thus, redesign of PIM37 alone was insufficient to reproduce the improved transformation phenotype observed with DesFusionPT37, suggesting that the combined PIM37 and linker redesign contributed to the improved performance of the fusion construct. Based on these results, DesFusionPT37 was selected for further characterization.

Drop assays demonstrated improved rescue of PT37‐mediated toxicity by DesFusionPT37 compared with the original FusionPT37 construct, and small‐scale expression experiments revealed detectable recombinant DesFusionPT37 production, which was not observed for FusionPT37 (Figure 5C,D). DesFusionPT37 was subsequently expressed in tBL21‐TEVP cells and purified using the optimized purification protocol (Figure 5E). SDS‐PAGE analysis demonstrated successful purification of PT37 (Figure 5F). Together, these results demonstrate that computational redesign of the PIM37‐containing fusion architecture converted a poorly productive PT37 fusion into a construct that supported recombinant protein production. Although these findings are consistent with improved productive toxin neutralization, they do not establish that the improved phenotype results from increased PT37–PIM37 binding affinity; changes in PIM37 stability, folding, or other properties of the redesigned fusion may also contribute.

### 3.8 Functional Validation of Purified PTwt Proteins

To determine whether the fusion‐based production strategy yielded correctly folded and enzymatically active proteins, we examined the nuclease activity of the purified PTs. We have previously demonstrated nuclease activity of PT7 using PT7 mutants that abolish in vivo toxicity but retain enzymatic activity in vitro (Nachmias et al. 2024). We therefore asked whether PTwt proteins produced using our workflow retained their enzymatic activity despite being purified as PT‐PIM complexes and then denatured and refolded.

Purified PT7, PT8 and PT9 were incubated with plasmid DNA and DNA degradation was analysed by agarose gel electrophoresis. DNase A served as a positive control. Consistent with our previous findings, purified PT7 exhibited robust nuclease activity, resulting in extensive degradation of the DNA substrate (Figure 6A). In addition to confirming the previously reported DNase activity of PT7, these experiments identified PT8 as a second nuclease within this family. In contrast, no detectable nuclease activity was observed for PT9 (Figure 6A).

**FIGURE 6:**
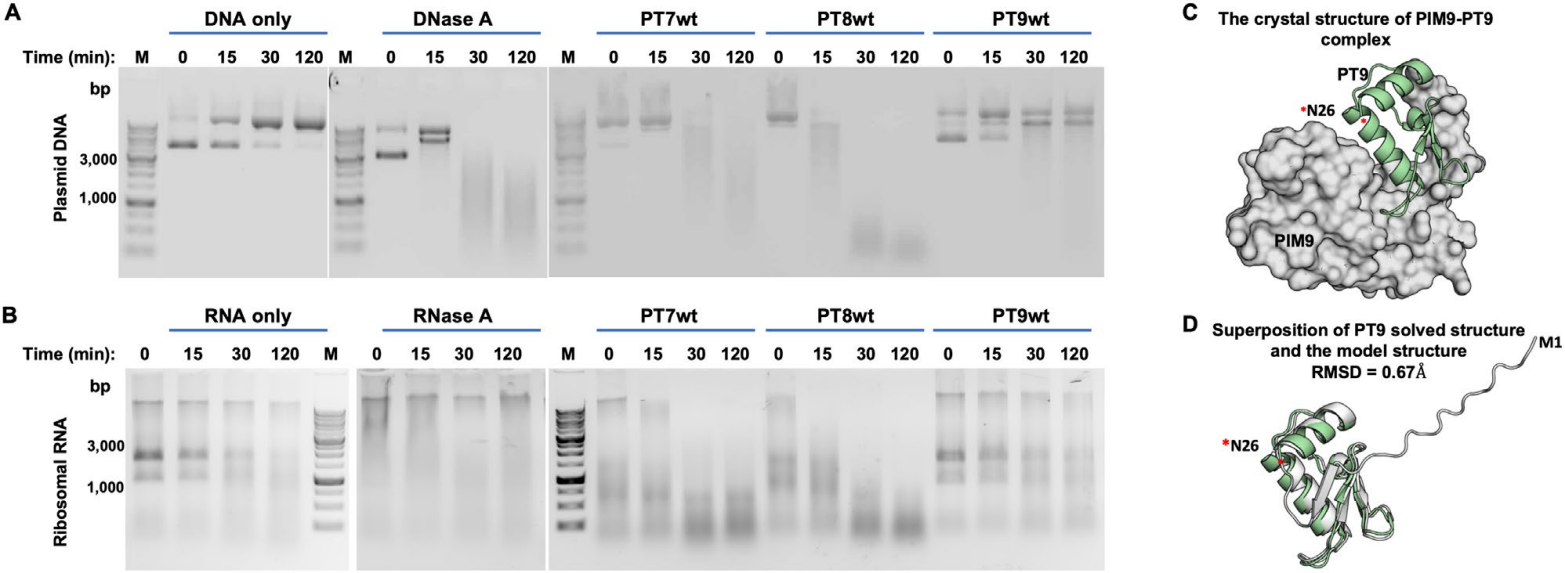
Functional and structural validation of purified wild‐type PTs. (A) In vitro nuclease activity assay of purified PT7, PT8 and PT9. Purified plasmid DNA was incubated with the indicated proteins for 0–120 min at 37°C and DNA degradation was analysed by 1% agarose gel electrophoresis. Buffer alone and DNase A served as the negative and positive controls, respectively. (B) In vitro RNase activity assay of purified PT7, PT8 and PT9. Purified ribosomal RNA was incubated with the indicated proteins under the same reaction conditions as in (A) and RNA degradation was analysed by agarose gel electrophoresis. (C) Crystal structure of the PIM9‐PT9 complex. PIM9 is shown as a white surface representation and PT9 is shown as a green cartoon representation. (D) Superposition of PT9 from the experimentally determined PIM9‐PT9 complex with the PT9 model, showing high overall structural similarity. Residues 1–25 at the PT9 N‐terminus were not modelled due to the absence of interpretable electron density. The Cα root‐mean‐square deviation (RMSD) between the experimental structure and the model is indicated.

To further investigate the substrate specificity of the purified toxins, we next examined whether they could degrade RNA. In our previous study, catalytically inactive PT7 mutants showed no detectable degradation of tRNA (Nachmias et al. 2024). However, its activity against other RNA substrates was not examined. Here, purified PT7, PT8 and PT9 were incubated with total RNA extracted from mammalian cells under identical reaction conditions, and RNA integrity was analysed by agarose gel electrophoresis. Both PT7 and PT8 efficiently degraded the RNA substrate, whereas PT9 showed no detectable RNase activity under the conditions tested (Figure 6B). These findings indicate that PT7 and PT8 possess both DNase and rRNase activities under the conditions tested, whereas PT9 showed no detectable nuclease activity towards either substrate.

Because PT9 lacked a positive functional readout, we used structural analysis to independently assess its folding following purification and refolding. PT9 purified using the denaturation and on‐column refolding procedure was incubated with independently expressed PIM9 to reconstitute the PT9‐PIM9 complex (Figure S5). The reconstituted complex was purified by affinity chromatography and SEC and subsequently crystallized. The crystal structure of the PT9‐PIM9 complex was determined at 1.5 Å resolution (Figure 6C; Table S1). Refolded PT9 adopted a well‐defined structure that closely matched the AlphaFold‐predicted model (RMSD 0.67 Å over 61 Cα atoms, Figure 6D). Moreover, its ability to re‐form a stable complex with its cognate PIM9 provides additional evidence that the refolded protein is structurally competent. Thus, although the molecular activity of PT9 remains unknown, these structural data demonstrate the recovery of a folded PT9 conformation following denaturation and refolding.

Together, these findings provide complementary functional and structural validation of the fusion‐based production and refolding platform. PT7 and PT8 retained nuclease activity following purification and refolding, whereas refolded PT9 recovered a well‐defined structure capable of re‐forming a complex with its cognate immunity protein. The successful recovery of functional PT7 and PT8 and structurally competent PT9 demonstrates that the platform can produce highly toxic proteins suitable for downstream biochemical, structural and mechanistic studies. Furthermore, identification of nuclease activity for PT8 illustrates how the platform enables functional characterization of previously inaccessible toxin families.

## 4 Discussion

The rapid emergence of AMR has intensified the search for antimicrobial agents with novel mechanisms of action (Antimicrobial Resistance 2022). The recombinant production of intrinsically toxic proteins has long represented a major challenge in protein engineering, structural biology and antimicrobial discovery. Although bacterial protein toxins are among the most biologically and therapeutically important examples, their inability to be produced has severely limited mechanistic investigation and translational development. In this study, we establish a modular production platform that overcomes this bottleneck through transient intramolecular neutralization during recombinant expression. By integrating toxin‐immunity fusion, computational protein engineering and streamlined purification, we successfully produced multiple previously intractable PTs while preserving their native enzymatic activity.

Multiple conventional recombinant expression strategies failed to produce detectable amounts of PTs (Figure 1). Neither tightly regulated inducible promoters, co‐expression of cognate immunity proteins, nor bacterial cell‐free expression systems enabled productive recombinant expression. These findings emphasize that the primary obstacle is not simply host viability but rather the rapid activity of newly synthesized toxin molecules during protein production. Even transient accumulation of a small fraction of unnaturalized toxin appears sufficient to prevent protein accumulation.

An interesting question raised by these findings is why recombinant co‐expression of toxin‐immunity pairs fails, even though these proteins are naturally co‐produced in their native bacterial hosts. Several non‐mutually exclusive explanations may account for this discrepancy. First, recombinant expression from multicopy plasmids under inducible promoters is likely to generate toxin molecules at rates that substantially exceed physiological levels, increasing the probability that toxin molecules transiently escape neutralization before immunity proteins can bind. Second, several bacterial toxins exhibit catalytic activity for which even a single active molecule may be sufficient to irreversibly inhibit essential cellular processes, thereby preventing continued protein synthesis and cell growth (Johnson et al. 2013). Third, PTs are typically synthesized via specialized secretory pathways and are frequently associated with dedicated secretion machinery, trafficking factors, or cellular scaffolds prior to export (Halvorsen et al. 2024). These native regulatory mechanisms are absent during heterologous expression in *E. coli* , potentially exposing the host cytoplasm to toxin molecules in a manner that does not occur under physiological conditions.

Our observation that bacterial cell‐free expression also failed to produce detectable toxin provides additional mechanistic insight into the production barrier (Figure S1C). Cell‐free protein synthesis has been widely used for recombinant production of toxic proteins because it eliminates host‐cell viability constraints while retaining the bacterial transcription‐translation machinery (Woelbern and Ramm 2024). The failure of PT expression under these conditions therefore suggests that cellular toxicity alone is not responsible for the production defect. Instead, newly synthesized toxin molecules may directly inhibit the transcription‐translation machinery present within the lysate. Because PT7 and PT8 exhibit DNase and RNase activities (Figure 6), they may rapidly degrade ribosomal RNA, messenger RNA, or other nucleic acid components required for continued protein synthesis, thereby terminating translation before detectable protein accumulates. Although this mechanism remains to be experimentally validated, our findings suggest that cell‐free expression may not overcome production barriers for toxins whose molecular targets are essential components of the cell‐free transcription‐translation machinery. Thus, the suitability of cell‐free expression for toxic proteins likely depends on the specific mechanism of toxicity.

The conceptual advance of this work lies in converting a naturally occurring toxin‐immunity interaction into a strategy for recombinant protein production. Rather than suppressing toxin expression or increasing host tolerance, our platform covalently links each toxin to its cognate immunity protein to suppress toxicity during biosynthesis, then releases and recovers the toxin during purification. The cognate PIMs were selected based on their natural association with the corresponding PTs and their experimentally demonstrated ability to rescue PT‐mediated toxicity, supporting toxin‐immunity neutralization as the basis for the fusion strategy.

Nevertheless, we cannot exclude the possibility that additional fusion‐dependent effects contribute to the protective phenotype. In principle, an N‐terminal fusion could reduce toxin activity by altering protein folding, oligomerization, localization, or accessibility of the catalytic site, or could influence recombinant production by changing mRNA stability or translation efficiency. To examine whether fusion to an unrelated soluble protein alone could reduce PT‐associated toxicity, we generated N‐terminal GFP fusions of PT7, PT8 and PT9. In contrast to the cognate PIM–PT fusions, transformation of the GFP–PT constructs into BL21(DE3) failed to yield viable transformants (Figure S1D). These results suggest that fusion to an unrelated soluble protein alone is not sufficient to reproduce the protective effect observed with the cognate PIM fusions. Moreover, toxicity‐deficient PT7 and PT8 mutants (Nachmias et al. 2024) could be detectably expressed whereas the corresponding wild‐type proteins could not, supporting toxicity rather than an intrinsic inability to express these protein scaffolds as a major production barrier. Together with the demonstrated ability of the cognate PIMs to rescue PT‐mediated toxicity, these observations support a major role for toxin‐immunity neutralization in enabling PT production, although contributions from altered folding, mRNA stability, translation, or other fusion‐dependent effects cannot be formally excluded.

An important aspect of the platform is its integration with modern computational protein modelling. Because none of the PT‐PIM systems examined here had experimentally determined structures, linker design relied entirely on deep learning‐based structure prediction. The agreement between computational predictions and experimental outcomes illustrates how recent advances in protein structure prediction can substantially accelerate construct optimization for previously uncharacterized protein families. More broadly, this study demonstrates how computational structural biology can be integrated directly into recombinant protein engineering workflows rather than being used solely for structural interpretation.

A second important advance is the incorporation of computational protein design into the production platform. Although fusion to native immunity proteins enabled the production of several PT families, the native PT37 fusion rescued toxicity but failed to accumulate to detectable levels, indicating that naturally evolved toxin–immunity pairs may not always be optimal for recombinant protein production. Importantly, redesign of the PT37 immunity protein and fusion architecture using ProteinMPNN (Figure 5) converted a nonproductive construct into one that supported recombinant expression and purification. This finding extends the platform beyond the direct use of naturally occurring toxin‐immunity pairs by demonstrating that computational redesign can improve the productive neutralization and production of challenging toxins. As computational protein‐design methods continue to improve, including approaches such as ProteinMPNN (Dauparas et al. 2022), PROSS (Weinstein et al. 2021), RFdiffusion (Watson et al. 2023) and related algorithms, optimization of toxin‐binding partners may further expand the applicability of this strategy to increasingly challenging toxin and antimicrobial protein families.

PT1 represents an important current limitation of the platform. In contrast to PT37, for which an intact fusion construct could be generated but failed to accumulate as recombinant protein, the major barrier for PT1 occurred already during molecular cloning and plasmid propagation. Repeated attempts to generate FusionPT1 constructs yielded very few transformants and led to frameshift mutations in the PT1 coding sequence, preventing evaluation of the fusion strategy at the expression level. Therefore, computational redesign of PIM1 was not pursued in the present study. Future strategies that further suppress toxin activity during cloning and plasmid propagation may be required before the fusion‐based production approach can be applied to exceptionally toxic proteins such as PT1. Nevertheless, the successful rescue of PT37 demonstrates that failure of a native toxin‐immunity fusion to support productive recombinant expression does not necessarily represent an intrinsic limitation of the platform and may, in some cases, be overcome through computational redesign of the toxin‐binding partner and fusion architecture.

Beyond improving expression, we also substantially simplified the purification workflow. Intracellular TEV‐mediated cleavage eliminated two purification steps while increasing protein yield approximately two‐ to four‐fold. This reduction in processing time, reagent consumption and production costs substantially improves the platform's scalability and practical utility for recombinant protein production, facilitating larger‐scale manufacturing for structural biology, biochemical characterization, high‐throughput screening and industrial biotechnology.

Importantly, the present study focuses on enabling recombinant production and functional characterization of intrinsically toxic proteins and does not address their activity following extracellular administration. The toxicity of the PTs examined here was demonstrated following intracellular expression, and whether the purified proteins can enter bacterial cells and exert antimicrobial activity when applied extracellularly remains unknown. For toxins acting on intracellular targets, such as the nuclease PT7 and PT8 proteins characterized here, extracellular antimicrobial activity would additionally require an effective mechanism for entry into target cells. Determining the delivery requirements and antimicrobial efficacy of the purified toxins will therefore be an important direction for future studies.

More broadly, we propose transient intramolecular neutralization as a general strategy for the recombinant production of intrinsically toxic proteins, including ones that were not evolved to directly serve as toxins. Although demonstrated here with PTs, the platform is conceptually applicable to a much broader range of intrinsically toxic proteins whose biological activity interferes with recombinant production. Future integration with advances in computational protein design may further expand this strategy beyond naturally occurring toxin‐immunity systems by enabling the engineering of synthetic neutralizing binders for proteins lacking cognate immunity partners. This work provides a scalable microbial biotechnology platform that should facilitate the recombinant production of previously inaccessible proteins for structural biology, synthetic biology, enzyme engineering and antimicrobial discovery.

## 5 Conclusion

In conclusion, we developed a modular strategy for the recombinant production of highly toxic bacterial proteins based on transient neutralization through fusion to cognate immunity proteins. This approach enabled the production and purification of three previously refractory PT families, while computational redesign extended the strategy to PT37 when the native toxin–immunity fusion was insufficient for productive expression. Integration of intracellular protease cleavage further simplified the purification workflow and improved protein recovery. Importantly, purified PT7 and PT8 retained nuclease activity following purification and refolding, whereas refolded PT9 recovered a well‐defined structure capable of re‐forming a complex, demonstrating that the platform can yield functionally active proteins suitable for downstream characterization. Together, these findings establish toxin–immunity fusion as an adaptable approach for overcoming toxicity‐associated barriers to recombinant protein production and provide access to previously intractable proteins for biochemical, structural and functional studies.

## Author Contributions

**Rina Fraenkel:** investigation, methodology, writing – original draft, visualization, validation. **Inbar Cahana:** investigation, writing – original draft, methodology, validation, visualization. **Tomer Sivan:** investigation, methodology. **Tal Fisher:** investigation, methodology. **Hen Nadav:** investigation, methodology. **Shani Bruchim:** investigation, methodology. **Jenna Weisz:** investigation, methodology. **Noam Deouell:** investigation, methodology. **Shani Cheskis:** investigation, methodology. **Maor Shalom:** investigation, methodology. **Lionel Imbert:** investigation. **Yaara Oppenheimer‐Shaanan:** investigation, methodology. **Asaf Levy:** investigation, writing – original draft, methodology. **Netanel Tzarum:** investigation, writing – original draft, methodology, validation, visualization, supervision.

## Funding

This work is funded in part by the Israel Innovation Authority, grants #81260 (to N.T.) and #81259 (to A.L.). The funding agencies had no role in study design, data collection, data analysis, interpretation of results, or manuscript preparation.

## Conflicts of Interest

The authors declare no conflicts of interest.

## Supporting information


**Figure S1:** Expression of the PTs using standard recombinant expression approaches. (A) Effect of wild‐type PT constructs on transformation efficiency in *E. coli* BL21(DE3). BL21(DE3) cells were transformed with pET28 constructs encoding PT1, PT7, PT8, or PT9, or with the empty pET28 vector as a control (emp). Colony numbers were determined following transformation and plating under identical conditions. No colonies were recovered for PT1, whereas PT7, PT8 and PT9 yielded markedly fewer colonies than the empty‐vector control. Data represent the mean ± SD of three independent transformations. (B) Recombinant expression of His‐tagged PT7, PT8 and PT9 from pBAD and pET28 vectors. Western blot analysis of small‐scale expression cultures showed no detectable PT protein. His‐tagged GFP served as a positive control. (C) Cell‐free expression of His‐tagged PT7, PT8 and PT9 using the pIVEX expression system. Western blot analysis revealed no detectable expression of the PTs. H23 was included as a positive expression control. (D) Effect of N‐terminal GFP fusion on PT‐associated transformation efficiency. BL21(DE3) cells were transformed with pET28 constructs encoding MBP‐GFP (control), GFP‐PT7, GFP‐PT8, or GFP‐PT9. Colony numbers were determined following transformation and plating under identical conditions. Whereas the MBP‐GFP control yielded transformants, no colonies were recovered for GFP‐PT7, GFP‐PT8, or GFP‐PT9. Data represent the mean ± SD of two independent transformations.
**Figure S2:** Structure‐guided design of FusionPT constructs. (A) Schematic representation of FusionPT constructs containing one to five GGGS linker repeats (FusionPT.0–FusionPT.4). (B). Structure‐guided design of FusionPT7, FusionPT8 and FusionPT9 constructs. Predicted structures of the native non‐covalent PT‐PIM complexes were superimposed with the corresponding FusionPT constructs containing GGGS linkers of varying lengths to evaluate preservation of the native PT‐PIM orientation. (C) Effect of linker length on rescue of PT9‐mediated toxicity. Drop assays of E. coli BL21(DE3) cells expressing FusionPT9.0–FusionPT9.4 under inducing conditions. The catalytically inactive PT9 K54A mutant served as a non‐toxic control. (D) Optimization of fusion protein expression conditions. SDS‐PAGE analysis of small‐scale expression of FusionPT7.2, FusionPT8.2 and FusionPT9.1 under different induction temperatures and expression times. Overnight expression produced the highest protein yield.
**Figure S3:** Construction of the transient tBL21‐TEVP strain. (A) Schematic illustration of the pBAD‐MBP‐TEVP expression plasmid and generation of the transient BL21(DE3) host used for intracellular TEV‐mediated cleavage. (B) SDS‐PAGE analysis of the purified PT9 following SEC indicating high purity.
**Figure S4:** Computational redesign of immunity proteins expands the fusion‐based production platform. (A) Western blot analysis of small‐scale expression of Flag‐tagged PT37 showing no detectable recombinant expression. Cell lysate expressing Flag‐tagged ERK was used as a positive control. (B) Summary of the FusionPT37 designs. Colony numbers were determined following transformation and plating under identical conditions. Data represent the mean ± SD of two independent transformations. (C) The amino acid sequences of the PIM and linker of the FusionPT37 designs. (D) Superposition of the predicted structure of PIM37 and the designed PIM37 on the predicted structure of FusionPT37 indicates a similar conformation of the PIM. (E) Structural comparison of the predicted FusionPT37 and DesFusionPT37 constructs. Superposition of the full fusion proteins (left) shows a similar overall conformation of the PT37‐PIM37 interface, with local changes in the linker conformation. Comparison of the PT37‐PIM37 interfaces (right) indicates that the redesigned construct preserves the overall toxin‐immunity interactions.
**Figure S5:** Purification of PIM9‐PT9 for crystallization. (A) Purification of the PIM9‐PT9 complex by Ni2+‐affinity chromatography. (B) SEC profile of the purified PIM9‐PT9 complex. The PIM9‐PT9 complex formed a dimer.
**Table S1:** Data collection and refinement statistics for the PIM9‐PT9 complex.

## Data Availability

The data that support the findings of this study are openly available in RCSB at https://www.rcsb.org/, reference number 33ZJ.
