# Supplementary material for: Modular Platform for Recombinant Production of Highly Toxic Bacterial Proteins
Source: Microb Biotechnol. 2026 Oct 16;19(10):e70453. doi: 10.1111/1751-7915.70453 (PMC13675652; doi:10.1111/1751-7915.70453)
Supplement: Supplementary file 1 [file MBT2-19-e70453-s001.docx]

**A modular platform for recombinant production of highly toxic bacterial proteins**

**Running title: Recombinant production of highly toxic proteins**

Rina Fraenkel^1,#^, Inbar Cahana^1,^^#^, Tomer Sivan^1^, Tal Fisher^1^, Hen Nadav^1^, Shani Bruchim^1^, Jenna Weisz^1^, Noam Deouell^1^, Shani Cheskis^2^, Maor Shalom^2^, Lionel Imbert^3^, Yaara Oppenheimer-Shaanan^2,4^, Asaf Levy^2^, Netanel Tzarum^1, 5*^

^1^ Department of Biological Chemistry, Alexander Silberman Institute of Life Sciences, Faculty of Mathematics & Science, The Hebrew University of Jerusalem, Jerusalem, Israel.

^2^ The Department of Plant Pathology and Microbiology, Robert H. Smith Faculty of Agriculture, Food, and Environment, The Hebrew University of Jerusalem, Rehovot, Israel.

^3^ Institut de Biologie Structurale, University of Grenoble, Grenoble, France.

^4^ Department of Life Sciences, Achva Academic College, Arugot, 79804, Israel.

^5^ The Center for Nanoscience and Nanotechnology, The Hebrew University of Jerusalem, Jerusalem, Israel.

^#^ These authors contribute equally

**^*^ Corresponding author:** Netanel Tzarum. Email; Netanel.tzarum@mail.huji.ac.il

**Supplementary Figure 1. Expression of the PTs using standard recombinant expression approaches.** **(A)** Effect of wild-type PT constructs on transformation efficiency in *E. coli* BL21(DE3). BL21(DE3) cells were transformed with pET28 constructs encoding PT1, PT7, PT8, or PT9, or with the empty pET28 vector as a control (emp). Colony numbers were determined following transformation and plating under identical conditions. No colonies were recovered for PT1, whereas PT7, PT8, and PT9 yielded markedly fewer colonies than the empty-vector control. Data represent the mean ± SD of three independent transformations. **(B)** Recombinant expression of His-tagged PT7, PT8, and PT9 from pBAD and pET28 vectors. Western blot analysis of small-scale expression cultures showed no detectable PT protein. His-tagged GFP served as a positive control. **(C)** Cell-free expression of His-tagged PT7, PT8, and PT9 using the pIVEX expression system. Western blot analysis revealed no detectable expression of the PTs. H23 was included as a positive expression control. **(D) Effect of N-terminal GFP fusion on PT-associated transformation efficiency.** BL21(DE3) cells were transformed with pET28 constructs encoding MBP-GFP (control), GFP-PT7, GFP-PT8, or GFP-PT9. Colony numbers were determined following transformation and plating under identical conditions. Whereas the MBP-GFP control yielded transformants, no colonies were recovered for GFP-PT7, GFP-PT8, or GFP-PT9. Data represent the mean ± SD of two independent transformations.

**Supplementary Figure 2. Structure-guided design of FusionPT constructs. (A)** Schematic representation of FusionPT constructs containing one to five GGGS linker repeats (FusionPT.0–FusionPT.4). (**B**). Structure-guided design of FusionPT7, FusionPT8, and FusionPT9 constructs. Predicted structures of the native non-covalent PT-PIM complexes were superimposed with the corresponding FusionPT constructs containing GGGS linkers of varying lengths to evaluate preservation of the native PT-PIM orientation. **(C)** Effect of linker length on rescue of PT9-mediated toxicity. Drop assays of *E. coli* BL21(DE3) cells expressing FusionPT9.0–FusionPT9.4 under inducing conditions. The catalytically inactive PT9 K54A mutant served as a non-toxic control. **(D)** Optimization of fusion protein expression conditions. SDS-PAGE analysis of small-scale expression of FusionPT7.2, FusionPT8.2, and FusionPT9.1 under different induction temperatures and expression times. Overnight expression produced the highest protein yield.

**Supplementary Figure 3. Construction of the transient tBL21-TEVP strain.** **(A)** Schematic illustration of the pBAD-MBP-TEVP expression plasmid and generation of the transient BL21(DE3) host used for intracellular TEV-mediated cleavage. (**B**) SDS-PAGE analysis of the purified PT9 following SEC indicating high purity.

**Supplementary Figure 4. Computational redesign of immunity proteins expands the fusion-based production platform.** **(A)** Western blot analysis of small-scale expression of Flag-tagged PT37 showing no detectable recombinant expression. Cell lysate expressing Flag-tagged ERK was used as a positive control. **(B)** Summary of the FusionPT37 designs. Colony numbers were determined following transformation and plating under identical conditions. Data represent the mean ± SD of two independent transformations. **(C)** The amino acid sequences of the PIM and linker of the FusionPT37 designs. **(D)** Superposition of the predicted structure of PIM37 and the designed PIM37 on the predicted structure of FusionPT37 indicates a similar conformation of the PIM. **(E)** Structural comparison of the predicted FusionPT37 and DesFusionPT37 constructs. Superposition of the full fusion proteins (left) shows a similar overall conformation of the PT37-PIM37 interface, with local changes in the linker conformation. Comparison of the PT37-PIM37 interfaces (right) indicates that the redesigned construct preserves the overall toxin-immunity interactions.

**Supplementary Figure 5.** Purification of PIM9-PT9 for crystallization. (**A**) Purification of the PIM9-PT9 complex by Ni^2+^-affinity chromatography. (**B**) SEC profile of the purified PIM9-PT9 complex. The PIM9-PT9 complex formed a dimer.

| **Data collection** | **PIM9-PT9** |  |  |  |
| --- | --- | --- | --- | --- |
| Beamline | ESRF ID-30A-3 |  |  |  |
| Wavelength (Å) | 0.96770 |  |  |  |
| Space Group | P6_1_22 |  |  |  |
| Unit cell parameters (Å,°) | a=51.5, b=51.5, c=273.5; 90,90,120 |  |  |  |
| Resolution range (Å) | 44.65-1.54 (1.59-1.54) ^a^ |  |  |  |
| Observations | 248,140 |  |  |  |
| Unique reflections | 32,956 (3,171) |  |  |  |
| Completeness (%) | 99.4 (100) |  |  |  |
| I/σ(I) | 13.9 (1.6) |  |  |  |
| CC_1/2_**^b^** | 0.99 (0.56) |  |  |  |
| R_merge_**^c^** | 0.07 (0.95) |  |  |  |
| R_pim_**^d^** | 0.04 (0.58) |  |  |  |
| Redundancy | 7.5 (6.7) |  |  |  |
| Refinement Statistics | |  |  |  |
| Resolution (Å) | 44.65-1.54 (1.59-1.54) |  |  |  |
| No. reflections | 32,942 |  |  |  |
| R_cryst_**^e^**/R_free_**^f^** | 0.227/0.244 |  |  |  |
| Number of atoms |  |  | |  |
| Protein | 1,653 |  |  |  |
| Water | 133 |  |  |  |
| Average B value (Å^2^) | | |  |  |
| All proteins | 25 |  |  |  |
| Water | 31 |  |  |  |
| R.m.s. deviation from ideal geometry | |  |  |  |
| Bond length (Å) | 0.006 |  |  |  |
| Bond angle (º) | 0.8 |  |  |  |
| Ramachandran Plot (%) **^g^** | |  |  |  |
| Favored | 98.5 |  |  |  |
| Outliers | 0.4 |  |  |  |
| PDB ID | 33ZJ |  |  |  |

**Table S1. Data collection and refinement statistics for the PIM9-PT9 complex.**

^a^ Parentheses refer to outer shell statistics.

^b^CC_1/2_ = Pearson Correlation Coefficient between two random half datasets.

^c^ R_merge_ = Σ*_hkl_* Σ*_i_* | *I_hkl,i_ - <I_hkl_>* | / Σ*_hkl_* Σ*_i_ I_hkl_*_,i_, where *I_hkl,i_* is the scaled intensity of the *i*^th^ measurement of

reflection *h, k, l*, and *< I_hkl_>* is the average intensity for that reflection.

^d^ R_pim_ = Σ*_hkl_* (1/(n-1))^1/2^ Σ*_i_* | *I_hkl,i_* - *<I_hkl_>* | / Σ*_hkl_* Σ*_i_ I_hkl,i_*, where n is the redundancy.

^e^ R_cryst_ = Σ*_hkl_* | *F_o_ - F_c_* | / Σ*_hkl_* | *F_o_* | x 100, where *F_o_* and *F_c_* are the observed and calculated structures factors.

^f^ R_free_ was calculated as for R*_cryst_*, but on a test set of 5% of the data excluded from refinement.

^g^ Calculated using MolProbity
